# Supplementary material for: A Tale of Two Seasons: Distinct Seasonal Viral Communities in a Thermokarst Lake
Source: Microorganisms. 2023 Feb 8;11(2):428. doi: 10.3390/microorganisms11020428 (PMC9964402; doi:10.3390/microorganisms11020428)
Supplement: Supplementary file 1 [file microorganisms-11-00428-s001.zip › microorganisms-2165408-supplementary.pdf]

Table S1. Results from the quast analysis of the assembly.

# contigs (>= 0 bp)	5 448 354
# contigs (>= 1000 bp)	602 394
# contigs (>= 5000 bp)	34 119
# contigs (>= 10000 bp)	9 938
# contigs (>= 25000 bp)	1 933
# contigs (>= 50000 bp)	382
Total length (>= 0 bp)	3.61E+09
Total length (>= 1000 bp)	1.35E+09
Total length (>= 5000 bp)	3.61E+08
Total length (>= 10000 bp)	1.99E+08
Total length (>= 25000 bp)	82 324 394
Total length (>= 50000 bp)	29 713 317
# contigs	2 115 508
Largest contig	362 592
Total length	2.36E+09
GC (%)	44.71
N75	710
L50	447 562
L75	1 110 151
# N's per 100 kbp	0

Table S2. Samples used for comparisons

Location	Type (subtype)	Depth	Season	Sequencing technology	Online availability	Authors
Lough Neagh	Water (Epilimnion)<br>Water (Epilimnion)	NA<br>NA	Summer<br>Winter	Illumina MiSeq	NCBI Bioproject PRJNA292054	Arkhipova et al., 2017
Stordalen Mire	Soil (Palsa)<br>Soil (Fen)<br>Soil (Bog)	0.01m to 0.85m<br>0.01m to 0.5m<br>0.01m to 0.33m	Summer<br>Summer<br>Summer	Illumina NextSeq	NCBI Bioproject PRJNA386568	Woodcroft et al., 2018
Bonanza Creek	Soil (Permafrost)<br>Soil (Palsa)<br>Soil (Bog)	0.3m to 0.7m	Summer<br>Summer<br>Summer	Illumina HiSeq	IMG/M ID Gs0063124	Hultman et al., 2015
Lake Croche	Water (Epilimnion)<br>Water (Epilimnion)<br>Water (Metalimnion)	0m<br>0m<br>3m	Winter<br>Summer<br>Summer	Illumina HiSeq	NCBI Bioproject PRJNA480789	Tran et al., 2018
Lake Simoncouche	Water (Epilimnion)<br>Water (Epilimnion)<br>Water (Metalimnion)	1m<br>1m<br>5m	Winter<br>Summer<br>Summer	Illumina HiSeq	NCBI Bioproject PRJNA480789	Tran et al., 2018

Table S3. Sample characteristics

Triplicate name	Sampling condition	Depth (m)	Temperature (°C)	Specific conductivity (μS/cm)	Oxygen (%)
2015 surface	Summer surface	0	12.75	44.4	45.6
2015 oxycline	Summer oxycline	0.5	11.95	45.2	9.2
2016 below the ice	Winter	Just below the ice	0.45	68.5	0
2017 surface	Summer surface	0.5	10.05	42.33	50.1
2017 bottom	Summer bottom	2.0	4.23	110.39	1.4

Table S4. Library characteristics

Sample	Triplicate	DNA used (ng)	Library preparation kit	Sequencing technology	Raw file size (reads)	Trimmed file size (reads)
2015 surface	1	1.0	Accel NGS 1S	Illumina HiSeq	13 633 701	13 438 653
	1	2.32	NEB Next Ultra	Illumina HiSeq	8 545 048	8 111 690
	2	1.0	Accel NGS 1S	Illumina HiSeq	5 291 007	5 215 055
	3	0.81	Accel NGS 1S	Illumina HiSeq	23 913 350	23 574 775
2015 oxycline	1	1.0	Accel NGS 1S	Illumina HiSeq	8 367 577	8 248 095
	1	3.02	NEB Next Ultra	Illumina HiSeq	8 062 946	7 837 543
	2	0.83	Accel NGS 1S	Illumina HiSeq	25 578 878	25 219 877
	2	9.96	KAPA Hyper Prep Kit	Illumina MiSeq	3 279 190	1 222 274
	3	1.0	Accel NGS 1S	Illumina HiSeq	14 387 116	14 182 824
2016 below the ice	1	1.0	Accel NGS 1S	Illumina HiSeq	12 338 461	12 159 565
	2	1.0	Accel NGS 1S	Illumina HiSeq	17 696 802	17 453 340
	3	1.0	Accel NGS 1S	Illumina HiSeq	15 126 049	14 902 871
2017 surface	1	1.0	Accel NGS 1S	Illumina HiSeq	15 209 956	15 000 826
	2	1.0	Accel NGS 1S	Illumina HiSeq	19 783 638	19 498 139
	3	1.0	Accel NGS 1S	Illumina HiSeq	16 900 776	16 660 469
2017 bottom	1	1.0	Accel NGS 1S	Illumina HiSeq	24 953 491	24 589 829
	2	1.0	Accel NGS 1S	Illumina HiSeq	19 892 594	19 608 039
	3	1.0	Accel NGS 1S	Illumina HiSeq	18 492 753	18 226 369

Table S5. Distribution of vOTUs containing integrase genes

Triplicate name	Number of vOTUs containing integrase	% of vOTUs containing integrase
2015 surface	24	3.10
2015 oxycline	25	3.16
2016 below the ice	25	3.14
2017 surface	25	2.52
2017 bottom	27	2.93

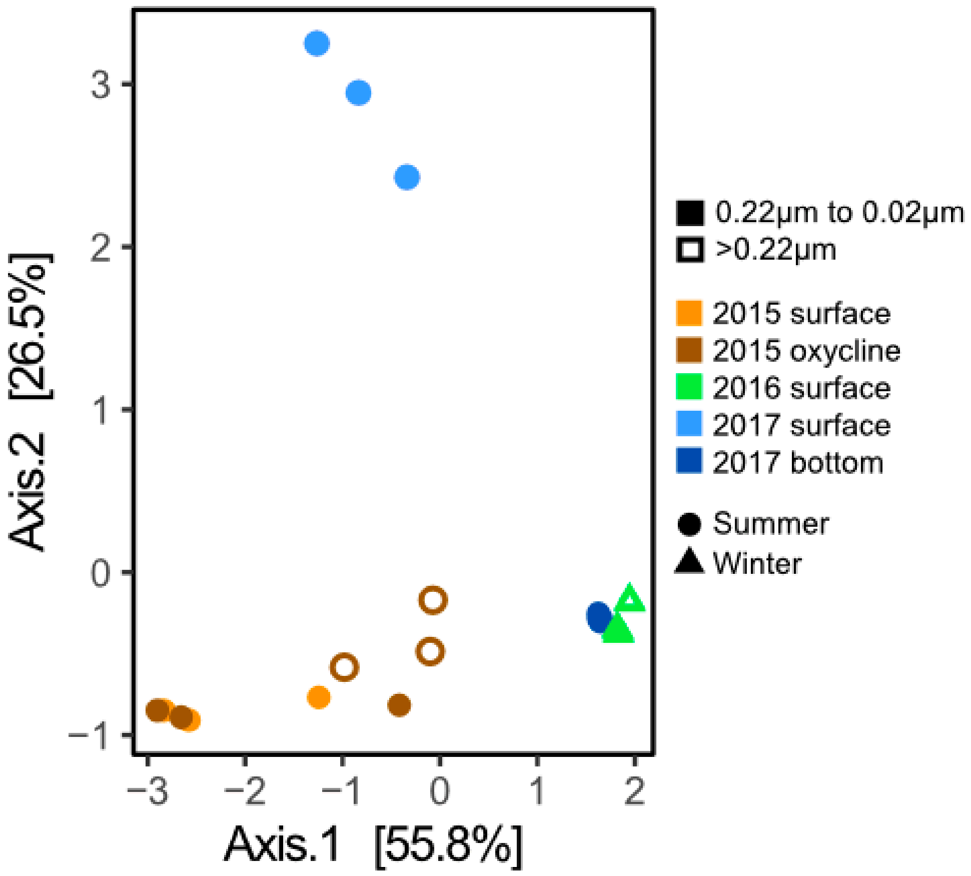

Figure S1. Representation of beta diversity of viral communities from the SAS 2A thermokarst lake in cellular (>0.22 µm) and viral enriched (0.22 µm to 0.02 µm) fractions. Beta diversity is represented using a principal coordinates analysis (PCoA) graph made using the Euclidean distance between viral communities.
